# Temporal relationship between hepatic steatosis and fasting blood glucose elevation: a longitudinal analysis from China and UK

**DOI:** 10.1186/s12889-024-19177-3

**Published:** 2024-07-12

**Authors:** Yujie Liu, Xian Liang, Yifan Hu, Ning Zhang, Xingren Zhu, Yuemei Feng, Zixiu Qin, Zihao Wang, Baima Kangzhuo, Xiong Xiao, Xing Zhao

**Affiliations:** 1 Department of Epidemiology and Biostatistics, West China School of Public Health and West China Fourth Hospital, Sichuan University, Chengdu, Sichuan, CN 610041 China; 2 Chengdu Center for Disease Control and Prevention, Chengdu, China; 3 School of Public Health, Kunming Medical University, Kunming, China; 4 School of Public Health, the Key Laboratory of Environmental Pollution Monitoring and Disease Control, Ministry of Education, Guizhou Medical University, Guiyang, China; 5 Chongqing Center for Disease Control and Prevention, Chongqing, China; 6 High Altitude Health Science Research Center of Tibet University, Lhasa, Tibet China

**Keywords:** Hepatic steatosis, NAFLD, Obesity, Type 2 diabetes

## Abstract

**Background:**

The link between nonalcoholic fatty liver disease and type 2 diabetes has not been fully established. We investigated the temporal relationship between nonalcoholic fatty liver disease (NAFLD) and type 2 diabetes (T2D), quantitatively assessed the impact, and evaluated the related mediation effect.

**Methods:**

This study involved participants from the China Multi-Ethnic Cohort Study and the UK Biobank. We performed cross-lagged path analysis to compare the relative magnitude of the effects between NAFLD and T2D using two-period biochemical data. Hepatic steatosis and fasting blood glucose elevation (FBG) represented NAFLD and T2D respectively. We fitted two separate Cox proportional-hazards models to evaluate the influence of hepatic steatosis on T2D. Furthermore, we applied the difference method to assess mediation effects.

**Results:**

In cross-lagged path analyses, the path coefficients from baseline hepatic steatosis to first repeat FBG (*β*_CMEC_ = 0.068, *β*_UK−Biobank_ = 0.033) were significantly greater than the path coefficients from baseline FBG to first repeat hepatic steatosis (*β*_CMEC_ = 0.027, *β*_UK−Biobank_ = -0.01). Individuals with hepatic steatosis have a risk of T2D that is roughly three times higher than those without the condition (*HR* = 3.478 [3.314, 3.650]). Hepatic steatosis mediated approximately 69.514% of the total effect between obesity and follow-up T2D.

**Conclusions:**

Our findings contribute to determining the sequential relationship between NAFLD and T2D in the causal pathway, highlighting that the dominant pathway in the relationship between these two early stages of diseases was the one from hepatic steatosis to fasting blood glucose elevation. Individuals having NAFLD face a significantly increased risk of T2D and require long-term monitoring of their glucose status as well.

**Supplementary Information:**

The online version contains supplementary material available at 10.1186/s12889-024-19177-3.

## Introduction

Nonalcoholic fatty liver disease (NAFLD) has emerged as a major public health issue worldwide. NAFLD is considered the leading cause of progression to more serious liver diseases [1, 2]. Notably, its global prevalence is around 25.24% [3] and still increasing [4], closely corresponding to the growing epidemics of obesity and type 2 diabetes (T2D) [5]. Some studies reported that 51.34% of patients with NAFLD also have obesity [3] and 22.51% of them concurrently suffer from T2D [6]. Consequently, numerous studies have focused on understanding the connections underlying this phenomenon to gain insight into the pathogenic mechanisms and treatments of NAFLD and T2D [7–9].

Despite the fact that obesity has been identified as the shared etiology of NAFLD and T2D [7, 8], the link between these above two diseases remains complex and the subject of ongoing debate. NAFLD was long thought to be a part of metabolic syndrome [10] and a long-term complication of T2D [9]. T2D could raise the risk of NAFLD developing into cirrhosis and even hepatocellular carcinoma [11]. One of the main mechanistic hypotheses supporting this view is that long-term exposure to high glucose levels could result in glucotoxicity. Lipotoxicity associated with insulin resistance in adipocytes and glucotoxicity have adverse effects on the growth of NAFLD [12]. Besides, The Framingham Heart Study provided epidemiological evidence that participants with confirmed T2D had a higher risk of NAFLD [13]. On the contrary, a growing viewpoint suggests that NAFLD is a multisystemic disease [14] and could precede T2D [15]. That is, NAFLD may also operate as a risk factor and a predictor of T2D [16]. The potential mechanism of this point is that ectopic fat accumulation in the liver, leading to increased hepatic glucose output, may raise the risk of T2D [7]. Several longitudinal studies suggested that participants with NAFLD at baseline had a higher incidence of T2D, although the results showed substantial variation across studies, with a rise in the incidence of up to 5.5-fold [13, 17, 18]. Additionally, evidence from Mendelian randomization studies has suggested a two-way relationship between NAFLD and T2D [19, 20].

And yet, most published research was unable to directly compare effect sizes in both directions, leaving the classical chicken-egg problem unresolved: which is the starting point, NAFLD or T2D [21, 22]? Or which is the dominant path between them? For longitudinal investigations involving multiple periods of biochemical data, the use of cross-lagged path analysis would probably be a more effective choice to answer the above questions. Cross-lagged path analysis could simultaneously assess the magnitude of two-way effects between interrelated variables in the same population by leveraging and analyzing multi-period continuous variables of biomarkers instead of binary disease outcomes [21]. In this model, the effect evaluation is standardized, allowing for a direct comparison of the magnitude of the effect in both directions. This scientific model has been successfully applied in multi-phase clinical trials and large population-based studies [23].

In this study, we included the participants from both the China Multi-Ethnic Cohort (CMEC) and UK Biobank in our analysis to account for the ethnic and geographical heterogeneity of NAFLD [16, 17]. We proposed to use cross-lagged path analysis to dissect the temporal relationship between NAFLD and T2D. Due to the requirements for continuous variables, we used hepatic steatosis and fasting blood glucose (FBG) elevation as early-stage indicators of NAFLD and T2D, respectively. Once the temporal sequence has been established, we further quantitatively assessed the impact of antecedent disease on subsequent disease using Cox-proportional hazard model to achieve a comprehensive understanding of their severity and to guide public health policies or clinical practice. Additionally, we constructed the mediation model for obesity (represented as the body fat percentage), hepatic steatosis, and T2D to examine the mediation effects.

## Materials and methods

### Study population

Launched in 2017, the China Multi-Ethnic Cohort Study has recruited 98,631 participants between the ages of 30 and 79 from southwest China. The CMEC collected multi-dimensional information on chronic diseases at baseline through face-to-face interviews with electronic questionnaires from the trained interviewers, physical examinations, and clinical laboratory testing. From 2020 to 2021, CMEC selected 10% of the baseline study participants to complete the first repeated survey by stratifying by region and conducting random cluster sampling with communities or villages as sampling units. The data collection of this repeated survey was the same as the baseline survey. Based on the comparison of baseline characteristics, the follow-up population is well-representative (see more details in the Appendix Table S1). Further information on the specifics of the CMEC study has been provided elsewhere [24].

Established in 2006, the UK Biobank has enrolled 502,392 participants between the ages of 40 and 79 from the United Kingdom. The UK Biobank collected comprehensive data from participants. Furthermore, participants were also regularly followed up to obtain health and disease-related data. More information about data collection has already been presented previously [25]. Since serum biochemical analyses were only performed at the baseline and the first repeat surveys, we limited the study sample to people from these two time periods.

In the current study, we primarily concentrated on individuals who had complete basic personal information and biochemical data at baseline and first repeat surveys. As body mass index (BMI) is closely linked to the target diseases and extreme BMI values raised our concerns about data accuracy for participants, we only included participants with a reasonable BMI (range from 14 to 45). Additionally, individuals taking insulin or other medications for diabetes, those with liver-related diseases (e.g., chronic hepatitis, liver cirrhosis), and individuals with a history of cancer were excluded from the study population. Finally, this study involved 7,668 subjects from the CMEC and 11,876 subjects from the UK Biobank. Figure S1 depicts the flow of participant selection. The strategies used in this research complied with the STROBE statement.

### Anthropometric information and biological samples

In the CMEC, trained doctors collected physical data at each site using uniform devices. We further calculated BMI and waist-to-hip ratio (WHR) using classic formulas. In addition, blood samples for testing were collected in the morning after participants had fasted for at least 12 h. These samples were then transported under cold chain to centralized regional laboratories for analysis. Plasma glucose and lipid profiles were evaluated using an AU5800 automated chemistry analyzer provided by Beckman Coulter Commercial Enterprise. Additionally, glycated hemoglobin (HbA1c) levels were measured with an MQ-6000 glycated hemoglobin analyzer from Shanghai Medconn Biotechnology Corporation. More details of the measurements of the blood parameters have been described in published articles [26]. Local CDCs were in charge of field QC, which included checking devices, ensuring study protocols and randomly selecting participants for re-examination.

In the UK Biobank, blood biochemistry biomarkers such as FBG, and triglycerides, were measured by Beckman Coulter AU5800 at baseline and the first repeat surveys. Proton density fat fraction (PDFF) was measured by magnetic resonance imaging at the second and third repeat surveys. Body fat percentage (BFP) was assessed by impedance measurement at each survey and we used this indicator to assess obesity. More details of study protocols have been described in published articles [25].

### Diagnostic criteria

In this study, we chose FLI and PDFF to diagnose hepatic steatosis. FLI, a frequently used surrogate marker in numerous studies, is a non-invasive and validated tool [27]. The variables used in the calculation of FLI are triglycerides, BMI, gamma-glutamyl transferase and waist circumference [28]. Further, hepatic steatosis was identified using FLI ≥ 60. PDFF is widely acknowledged as a reliable indicator for estimating liver fat content [29, 30]. When using PDFF for analysis, we determined hepatic steatosis based on PDFF values exceeding 5.6% [29].

In addition to using FBG as the marker of diabetes, we also recognized T2D through self-reported history, FBG ≥ 7.0mmol/L, or hemoglobin A1c ≥ 6.5% on physical examination.

We detected other chronic diseases based on personal disease history with the diagnostic record, or the results of physical examination and serum biochemical analyses. The latter criteria were: (1) for hypertension: systolic blood pressure ≥ 130 mmHg, or diastolic blood pressure ≥ 80 mmHg; (2) for dyslipidemia: TC ≥ 6.22 mmol/L, LDL ≥ 4.14 mmol/L, TG ≥ 2.26 mmol/L or HDL ≤ 1.04 mmol/L.

### Questionnaire survey and covariates selection

Personal information was obtained by completing a comprehensive electronic questionnaire. Based on the information mentioned above and the existing literature [13, 31], we selected sex, age, WHR, ethnic group, occupation (CMEC) or deprivation index (UK Biobank), education, cigarette smoking, alcohol status, dietary score, and non-sedentary physical activity (METs-h/day) as covariates for the subsequent analysis. Considering that the composition of the FLI consists of BMI, we used WHR instead of BMI to assess obesity. Dietary score was calculated by the Dietary Approaches to Stop Hypertension (DASH) score [32] in CMEC and the healthy diet pattern score [33] in UK Biobank. Table S2 displays the descriptions of covariates in detail.

### Statistical analysis

At the start of this study, we described the population characteristics at baseline and the first repeat survey for both cohorts. We used the median [interquartile range] for continuous variables and numbers (percentages) for categorical variables as statistical descriptive indicators.

We conducted cross-lagged path analysis to analyze the directional link between FLI and FBG that have been measured repeatedly at 2-time points. At first, we conducted the regression residual analysis to adjust the FLI and FBG indices at baseline and the first repeat assessment by previously mentioned confounders and used Z-transformation to standardize residuals. Then we applied structural equation modeling to perform the cross-lagged path analyses. We could determine the temporal sequence relationship through comparing the path coefficients *β*_1_ (baseline FLI to subsequent FBG) and *β*_2_ (baseline FBG to subsequent FLI). Fisher’s *Z* test was used to examine the difference between *β*_1_ and *β*_2_. We selected the comparative fitness index (CFI) and standardized root mean square residual (SRMR) to measure the goodness of models. CFI ≥ 0.95 and SRMR ≤ 0.08 implied that the model fitted the sample data well. We also conducted stratified analyses to examine potential effect heterogeneity among the predefined stratification subpopulations, which included sex, age (60 years as the cut-off value), ethnic group, whether suffering from hypertension, and whether suffering from hyperlipidemia. Additionally, a sensitivity analysis was performed in the current study. We further applied cross-lagged path analysis to a population that had neither NAFLD nor T2D at baseline.

After determining the main direction of effects between the two conditions, we focused on the magnitude of the impact of antecedent disease on subsequent disease by fitting the Cox proportional-hazards models and adjusting for confounders mentioned earlier. Then we constructed a regression-based mediation model to investigate the related mediation effects among obesity, hepatic steatosis and T2D. We fitted two separate Cox proportional-hazards models to assess the association between exposure-outcome, as well as exposure-mediator-outcome, adjusted for previously mentioned confounders. We calculated the total, direct, indirect effects and the proportion of mediation using the difference method [34]. Then we used the bootstrap method to compute the 95% confidence intervals (*CI*). The proportion of mediation was calculated by (ln (*HR*_Tot_)- ln (*HR*_DE_)) / ln (*HR*_Tot_). Because only the UK Biobank had the data of long-term follow-up outcome and BFP, survival and mediation analyses were limited to this cohort. Figure S2 and S3 show the specific flow of participant selection.

Furthermore, we conducted several validation analyses to demonstrate the reliability of FLI. We used PDFF instead of FLI for analyses and compared their results. The lack of concurrent PDFF and FBG data limited our capacity to conduct cross-lagged path analyses with PDFF. Hence, we only replicated the survival and mediation analyses using PDFF, following the same procedures as before. All models satisfied the proportional hazards assumption (Figure S4 - S6) and had no interaction between exposure and mediator (Table S3 and S4).

For all of the above analyses, we used multiple imputation to deal with missing data. The dataset was filled five times independently. Each complete dataset was analyzed separately to generate five estimates, which were then pooled using Rubin’s rules to produce the final result. We performed all statistical analyses in R 4.1.0. For convenience, the baseline, first repeat and second repeat assessments were referred to as T1, T2 and T3 in the following content.

## Results

### Participant characteristics

Table 1 summarizes our study population characteristics at T1 and T2 from CMEC and UK Biobank. In CMEC, we included 7,668 subjects with a median age at T1 of 50[43.00, 58.00] years, 63.11% of females, and a median follow-up between T1 and T2 of 1.98 [1.78, 2.16] years. The median FBG and FLI at T1 were 5.08 [4.71, 5.43] mmol/L and 22.33 [9.32, 47.30], respectively. Compared to the characteristics at T1, the number of smokers and drinkers, as well as non-sedentary physical activity, reduced while FLI increased at T2. In UK Biobank, we included 11,876 subjects with a median age at T1 of 58.00 [52.00, 63.00] years, 48.15% of females, and a median follow-up between T1 and T2 of 4.43 [2.11, 6.12] years. The median FBG and FLI at T1 were 4.88 [4.55, 5.23] mmol/L and 41.63[18.19,70.63], respectively. Compared to the characteristics at T1, the number of smokers and drinkers reduced while FLI increased at T2. In comparison to CMEC, the UK Biobank population had a lower proportion of females, older age, higher BMI and FLI, a greater proportion of higher educated people and regular drinkers, as well as less non-sedentary physical activity.

**Table 1:** Characteristics of participants at T1 and T2 from the CMEC and the UK Biobank ^a^

	CMEC (*N* = 7,668)		UK Biobank (*N* = 11,876)		
	T1	T2	T1	T2	
Characteristic					
Age, years	50.00 [43.00, 58.00]	52.00 [45.00, 60.00]	58.00 [52.00, 63.00]	63.00 [56.00, 67.00]	
Body mass index, kg/m [2]	24.06 [21.83, 26.38]	24.33 [22.15, 26.77]	26.24 [23.85, 29.08]	26.29 [23.91, 29.17]	
Waist-to-hip ratio	0.88 [0.84, 0.93]	0.89 [0.84, 0.94]	0.87 [0.80, 0.93]	0.88 [0.82, 0.94]	
Female Sex, n (%)	4839 (63.11)		5718(48.15)		
Ethnic group, n (%)					
Majority (Han / White)	4275 (55.75)		11605 (97.72)		
Minority (Others)	3393 (44.25)		271 (2.28)		
Education, n (%)					
Less than high school	3964 (51.70)		1059 (8.92)		
High school education	2873 (37.47)		5647 (47.55)		
Higher education	830 (10.82)		5116 (43.08)		
**Lifestyle factors**					
Cigarette smoking, n (%)					
Current	1361 (17.75)	1233 (16.08)	769 (6.48)	533 (4.49)	
Alcohol status ^c^, n (%)					
Occasional drinker	2196 (28.64)	1626 (21.20)	2280 (19.20)	2518 (21.20)	
Regular drinker	921 (12.01)	822 (10.72)	8971 (75.54)	8596 (72.38)	
Non-sedentary physical activity ^b^, METs-h/day	22.89 [12.60, 37.89]	18.00 [8.90, 31.80]	4.14[1.89, 8.06]	4.23[2.02, 7.90]	
Dietary score ^d^	21.00 [18.00, 24.00]	21.00 [18.00, 24.00]	4 [3, 5]	4 [3, 5]	
**Main measures**					
Fast blood glucose, mmol/L	5.08 [4.71, 5.43]	4.93 [4.61, 5.30]	4.88 [4.55, 5.23]	4.93 [4.62, 5.26]	
Fatty liver index	22.33 [9.32, 47.30]	25.80 [10.82, 51.77]	41.63[18.19,70.63]	43.65[20.79,71.18]	
Body fat percentage	—	—	29.60 [24.00, 36.10]	30.30 [24.80, 36.80]	

^a^ Median [interquartile range] or counts (proportion). Due to the presence of missing data, proportions may not add up to 1

^b^ Because CMEC had a large proportion of people working in agriculture and animal husbandry, the non-sedentary physical activity value was higher than people in UK Biobank

^c^ Drinking less than three times a month was considered an occasional drinker, and drinking more than once a week was considered a regular drinker

^d^ Dietary score referred to the DASH score in CMEC, and healthy diet pattern score in UK Biobank

### Cross-lagged path analysis

**Fig. 1:**
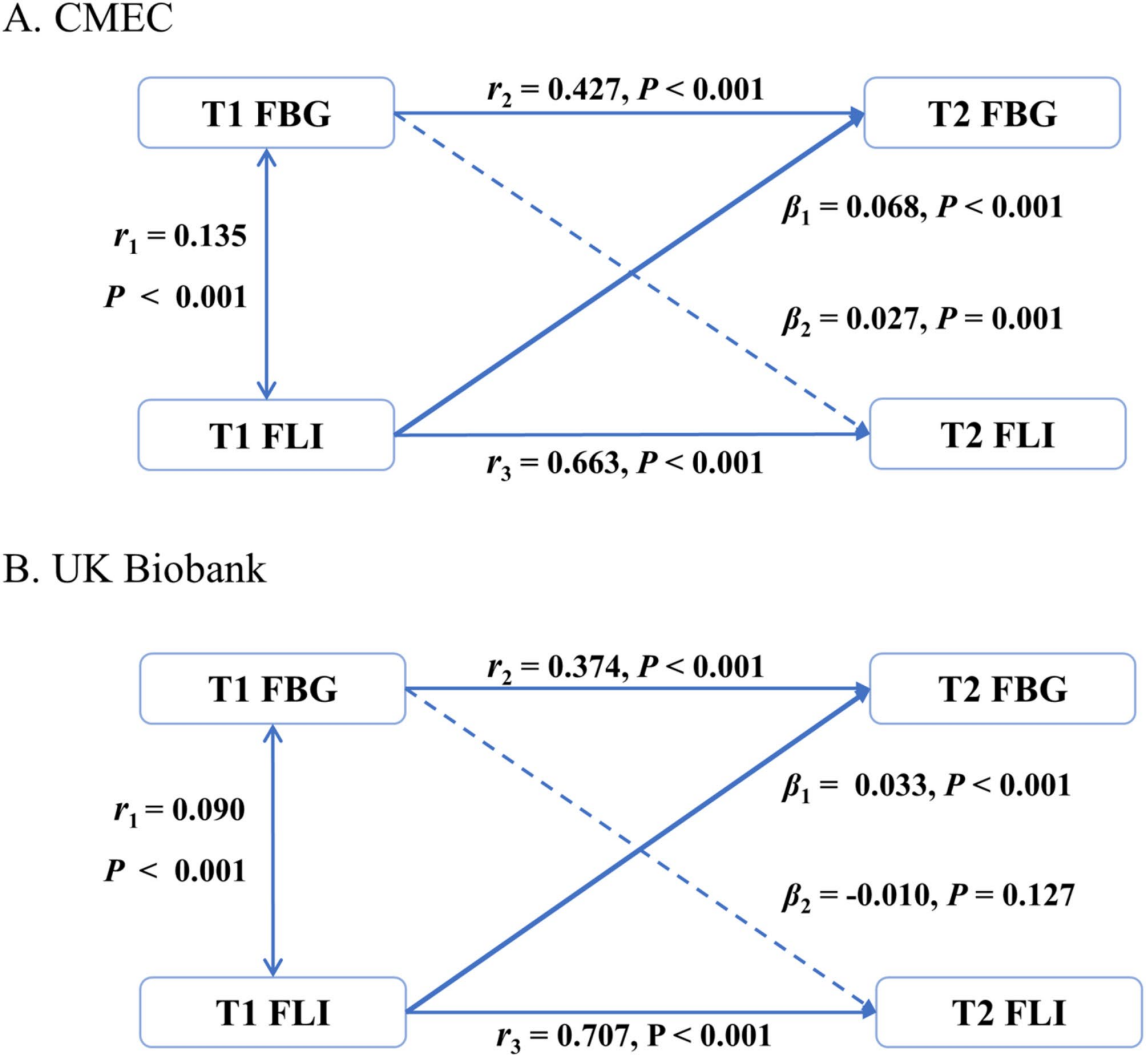
Cross-lagged path analysis of the FLI with FBG. *β*_1_ indicates the coefficients of the path from T1 FLI to T2 FBG; *β*_2_ indicates the coefficients of the path from T1 FBG to T2 FLI. The conditional correlation coefficient between T2 FBG and T2 FLI was set to zero. The covariates adjusted in the model include sex, age, WHR, ethnic group, occupation (CMEC) or deprivation index (UK Biobank), education, cigarette smoking, alcohol status, dietary score, and non-sedentary physical activity. Abbreviation: FLI: the fatty liver index; FBG: fast blood glucose

Figure 1 depicts the results of cross-lagged path analyses. In CMEC, the coefficient of the path from T1 FLI to T2 FBG (*β*_1_ = 0.068, *P* < 0.001) was approximately 2.5-fold that of the coefficient of the path from T1 FBG to T2 FLI (*β*_2_ = 0.027, *P* = 0.001). The difference between two path coefficients (*β*_1_ and *β*_2_) was statistically significant (*Z* = 3.047, *P* = 0.002). The variance (*R*^*2*^) of T2 FLI was 0.44 and 0.20 for T2 FBG. This model fitted well with CFI and SRMR of 0.983 and 0.021, respectively. In UK Biobank, the coefficient of the path from T1 FLI to T2 FBG (*β*_1_ = 0.033, *P* < 0.001) was approximately 3-fold that of the coefficient of the path from T1 FBG to T2 FLI (*β*_2_ = -0.01, *P* = 0.127). The difference between two path coefficients was statistically significant (*Z* = 3.771, *P* < 0.001). The variance (*R*^*2*^) of T2 FLI and FBG were 0.50 and 0.14, respectively. This model also had an equally good fit with CFI and SRMR of 0.995 and 0.012. The results from both cohorts indicated that the dominant path was the one from T1 FLI to T2 FBG.

**Fig. 2:**
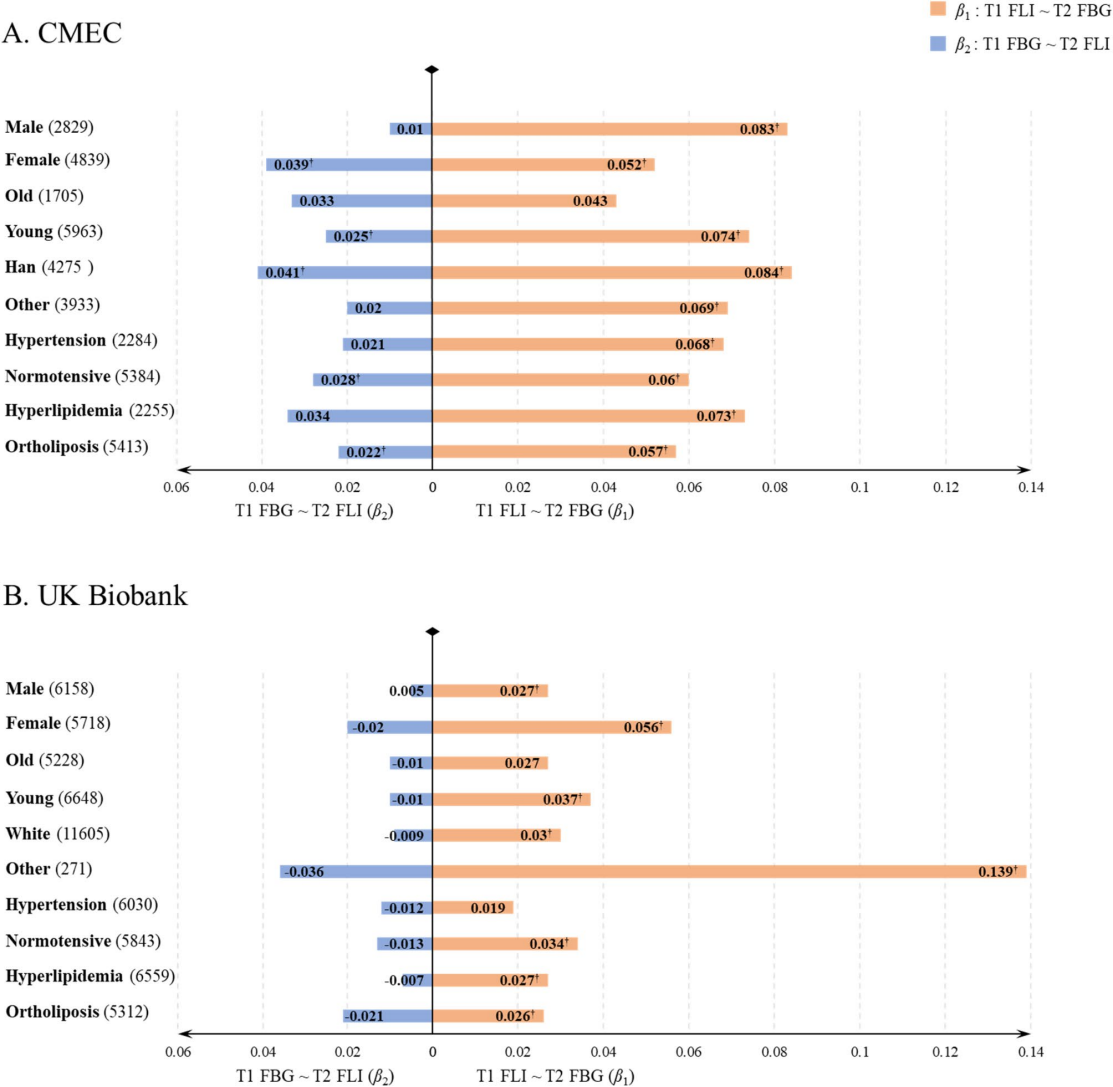
Cross-lagged path analysis of FLI with FBG in subgroups. Subgroups include male / female, old / young, whites / others, hypertension / normotensive, and hyperlipidemia / ortholiposis. The X-axis represents the magnitude of path coefficients in two directions. To the right are the path coefficients from T1 FLI to T2 FBG, and to the left are those from T1 FBG to T2 FLI. The length of the bars only indicates the absolute value of the coefficients, with the specific coefficient values displayed within the bars. ^a^ Numbers in brackets show the number of participants in each group. § represented *P* < 0.05. The covariates adjusted in the model include sex, age, WHR, ethnic group, occupation (CMEC) or deprivation index (UK Biobank), education, cigarette smoking, alcohol status, dietary score, and non-sedentary physical activity. Abbreviation: FLI: the fatty liver index; FBG: fast blood glucose

Figure 2 displays the cross-lagged path analyses results in predefined stratification subpopulations. These results were generally consistent with the results of the whole population, although the effects were weak in some subgroups. Notably, the difference between two path coefficients in the hypertension group was not statistically significant in either cohort

Table S5 (See appendix for details) shows the cross-lagged path analyses results in the limited population (CMEC: *n* = 6,327; UK Biobank: *n* = 8,335). Consistent with the previous results, the path from T1 FLI to T2 FBG remained dominant with statistically significant differences between two path coefficients in both cohorts (*Z*_CMEC_ = 2.674, *P* = 0.008; *Z*_UK Bio−bank_ = 2.941, *P* = 0.003).

### Survival analysis

Table 2 displays the results of Cox-proportional hazard models for T1 FLI or T3 PDFF and follow-up T2D. The findings of FLI were predominantly in line with the findings of PDFF. With the increase in FLI, the risk of developing T2D increased. The *HR* for the continuous FLI was 1.034 (1.033, 1.035), for standardized was 2.709 (2.628, 2.791), and for binary FLI (≥ 60 compared to < 60) was 3.478 (3.314, 3.650). For per unit increase in PDFF, the risk of developing T2D was elevated by 0.080-fold [*HR*: 1.080 (1.056, 1.105)]. The *HR* for the standardized PDFF was 1.427 (1.285, 1.585), for binary PDFF (> 5.6% compared to ≤ 5.6%) was 2.455 (1.661, 3.628).

**Table 2:** Cox-proportional hazard model for T1 FLI or T3 PDFF and follow-up type 2 diabetes ^a^

	FLI			PDFF		
	*HR*	95%*CI*	*P*	*HR*	95%*CI*	*P*
Continuous	1.034	(1.033, 1.035)	< 0.001	1.080	(1.056, 1.105)	< 0.001
Standardized ^b^	2.709	(2.628, 2.791)	< 0.001	1.427	(1.285, 1.585)	< 0.001
Binary	3.478	(3.314, 3.650)	< 0.001	2.455	(1.661, 3.628)	< 0.001

*Abbreviation* FLI: the fatty liver index; PDFF: proton density fat fraction; *HR*: hazard ratio; *CI*: confidence interval

^a^. The covariates adjusted in three models include sex, age, WHR, ethnic group, deprivation index, education, cigarette smoking, alcohol status, dietary score, and non-sedentary physical activity

^b^. This result is interpreted as the effect of 1 SD change in the FLI or PDFF on outcome

### Mediation analysis

**Fig. 3:**
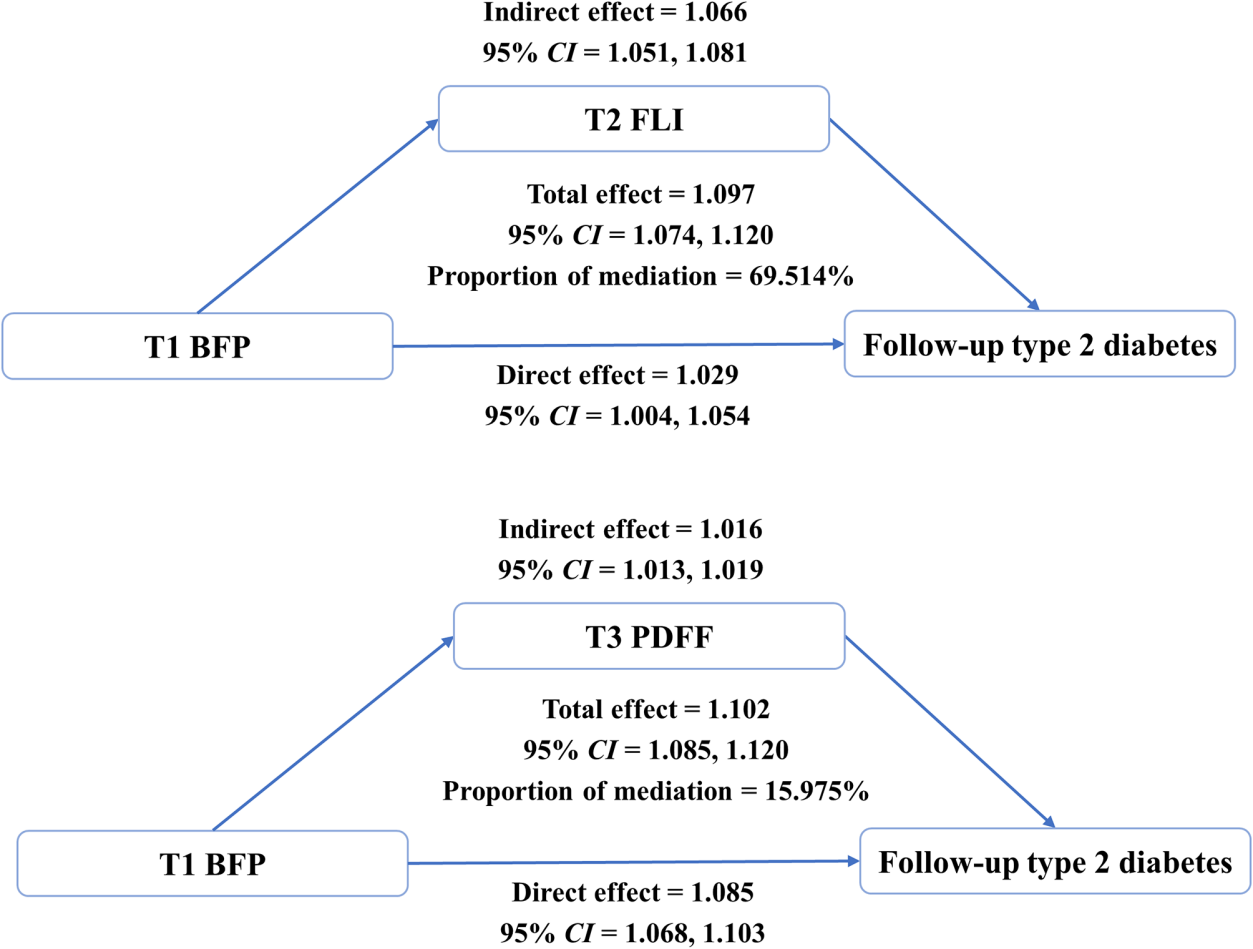
Mediation model for T1 BFP, T2 FLI or T3 PDFF, and follow-up type 2 diabetes. The covariates adjusted in the model include sex, age, WHR, ethnic group, deprivation index, education, cigarette smoking, alcohol status, dietary score, and non-sedentary physical activity. Abbreviation: BFP: body fat percentage; FLI: the fatty liver index; PDFF: proton density fat fraction; CI: confidence interval

We further excluded individuals who had T2D before T1, and 11,627 participants from the UK Biobank were involved in the mediation analyses. During the median follow-up of 14.195 [13.134, 14.745] years, 478 (4.11%) participants developed T2D and 417 participants died after the baseline survey. The total person-years of follow-up was 159075.6. Figure 3 presents the mediation model for T1 BFP, T2 FLI or T3 PDFF, and follow-up T2D. For FLI, the total effect was represented by Hazard Ratio (*HR* = 1.097, 95% *CI* = 1.074,1.120) from the exposure-outcome Cox proportional-hazards model. The indirect-effect *HR* (1.066 [1.051,1.081]) was greater than the direct-effect *HR* (1.029 [1.004,1.054]). The proportion of mediation was estimated to be 69.514%. For PDFF, the total effect *HR* was 1.102 [1.085,1.120]. The indirect-effect *HR* was 1.016 [1.013,1.019] and the direct-effect *HR* was 1.085 [1.068,1.103]. The proportion of mediation was estimated to be 15.975%. All of these findings indicated that hepatic steatosis appears to be a significant mediator in the link between BFP and T2D.

## Discussion

In two longitudinal cohort studies with multiple periods of biochemical data, we examined the temporal relationship between hepatic steatosis and FBG elevation. Our cross-lagged path analyses suggest that the development of hepatic steatosis precedes the development of FBG elevation, with both cohorts showing a consistent relationship. The stratified analyses suggest that the association between hepatic steatosis and FBG elevation disappeared in the hypertension group. Additionally, the survival analyses suggest that individuals with hepatic steatosis have a significantly higher risk of T2D. Further, our mediation analyses suggest that hepatic steatosis mediated a part of the total effect between obesity and follow-up T2D.

The current study confirms that the dominant path was the one from T1 FLI to T2 FBG. Several previous observational researches have found the significant link between NAFLD and a high risk of T2D [17, 35]. Yamazaki et al. also demonstrated that improvements in NAFLD could reduce the incidence of T2D. While these studies support our findings, they only focused on the unidirectional effects of NAFLD on T2D by using binary variables for diseases and calculating adjusted hazard ratio or adjusted odds ratio. Meanwhile, some studies have also suggested NAFLD as a complication of T2D. The evidence from cross-sectional studies [6] indicated that there is a high prevalence of NAFLD among patients with T2D. Ma et al. [13]. conducted a parallel analysis of the link between baseline fatty liver and incident T2D, as well as baseline fasting plasma glucose and incident fatty liver based on around 1000 participants from the Framingham Heart Study cohort. This study indicated a two-way relationship between liver fat and T2D.Notably, several studies using Mendelian randomization analysis also indicated the two-way relationship between them. Liu et al. suggested NAFLD could be divided into two subtypes. “Nature” NAFLD increase the risk of type 1-like diabetes. Meanwhile, T2D could increase the risk of “nurture” NAFLD [19]. De Silva et al. implied insulin resistance (IR) promote the emergence of NAFLD and NAFLD is associated with an increased risk of developing T2D [20]. The findings of these bi-directional researches for each direction cannot be directly compared due to the use of different indicators to assess NAFLD and T2D.

The stratified cross-lagged path analysis in the hypertension group implies that there is likely to be a potential mediator related to the cause of hypertension in the NAFLD-T2D pathway. Previous evidence indicated that NAFLD might be a cause of hypertension [36]. Meanwhile, it has been demonstrated that hypertension and diabetes are bad companions and often coexist, with related pathological mechanisms between the two [37], such as the nitrous oxide pathway of IR and the subsequent stimulatory effect on the sympathetic excitation, growth of smooth muscle, and sodium-fluid retention. It is reasonable to speculate that the common cause of hypertension and diabetes may be the mediator in the FLI-FBG path. Controlling hypertension, as a descendent of the mediator, could block the causal path from T1 FLI to T2 FBG.

The survival analyses show that in European populations, individuals with hepatic steatosis have a risk of T2D that is roughly three times higher than those without the condition. Results from prior studies have varied widely, with risks ranging from a 33% increase [38] to a 5.5-fold increase [39]. Differences in these results may be attributed to variances in geographical regions and the duration of follow-up. Moreover, the findings of FLI are consistent with the PDFF findings, providing the confirmation of the reliability of FLI. There have been various potential mechanisms proposed to elucidate the complex pathogenesis of how NAFLD leads to T2D [18, 22]. In patients with NAFLD, ectopic fat accumulation in the liver may increase hepatic glucose output, which could adversely affect glucose metabolism. Additionally, molecules associated with liver inflammation that are secreted by the liver, such as angiopoietin-like protein, is a risk factor for T2D. Further research indicates that fatty liver exhibits distinct endocrine functions compared to healthy liver tissue. A fatty liver can differentially express and secrete various proteins (hepatokines) into the circulation, such as Fetuin-A, ANGPTL3, FGF21, Selenoprotein P, Fetuin-B, and Follistatin. These factors can adversely affect the development and progression of T2D [40].Increased levels of total serum bile acids, diacylglycerols, and ceramides are also potential risk factors.

The results of the mediation analyses reveal a significant and critical role of hepatic steatosis in the association between obesity and T2D. It highlights the importance of obesity-NAFLD-T2D underlying pathophysiological and metabolic mechanisms. Few studies have previously investigated this mediation effect in this complex link, so we lack data available for comparison. Obesity may cause NAFLD through two main mechanisms. The primary cause of liver injury [41] is due to impaired suppression of lipolysis and increased free fatty acids (FFAs) release in obesity. An increase of de novo lipogenic pathways within hepatocytes would also promote hepatic steatosis [9]. The potential mechanisms by which NAFLD causes T2D have been discussed previously.

The prevalence of obesity and NAFLD are expected to increase globally. It is anticipated that these trends have an adverse effect on the prevalence of T2D. Our study suggests that identifying and managing hepatic steatosis in individuals may be an important preventive strategy for reducing the risk of developing T2D, particularly in the initial phases of the condition. This result reinforces the causal relationship between NAFLD and T2D [22], highlighting the importance of hepatic steatosis as a potential contributor to the development of T2D. High-risk groups should be prioritized for the prevention of T2D, and the management of obesity is still the priority. Future analyses should consider the heterogeneity in obesity and T2D [42], and aim to provide important new insights into the impact of metabolically unhealthy obesity on the risk of T2D.

### Strength and limitation

The present study shows several strengths. Unlike previous studies primarily focused on Asian populations, we used two large cohorts from China and the UK to explore the relationship between liver fat and T2D in varying ethnic groups, which can provide new population-based evidence and expand on existing information. Further, we analyzed multi-period biochemical data using cross-lagged path analysis, a more effective approach to detective the temporal sequence relationship between inter-related variables. As far as we know, this is the initial research to compare the relative magnitude of the effects between hepatic steatosis and fasting blood glucose elevation. Notably, previous studies have rarely estimated mediation effect to measure the contribution of liver fat to the obesity-T2D pathway. By performing mediation analyses, our study provides population-based insights for exploring the complete mechanistic pathway.

We acknowledge that the current study had several limitations. First, we only included those who completed both the baseline and first repeat survey, so some selection bias may exist and our study population cannot represent the entire population. Additionally, our study excluded individuals taking insulin or other medications for diabetes, so the conclusions cannot be generalized to the population of patients with advanced diabetes. Second, due to the lack of concurrent data for PDFF and FBG, the PDFF data did not support its application in cross-lagged analyses. We were constrained to perform these analyses using FLI, which is the practical indicator in large general population cohorts. However, FLI is not the best estimate for hepatic steatosis. Moreover, as FLI includes BMI in its composition, there is a possibility of overestimating the mediation effect. Third, in cross-lagged path analyses, we could not distinguish the type of diabetes. The participants we included were all over the age of 30 years, among whom the prevalence of type 1 diabetes (T1D) was low, at 0.69 per 100,000 person-years in China and lower in the UK. Thus, we anticipated that the sample size of T1D patients would be very small in this study and that the failure to distinguish between types of diabetes would not result in significant bias. Finally, in assessing mediation effects, we did not consider the impact of heterogeneity in obesity and T2D. The current mediation analysis is exploratory and will need to be further investigated in the future.

## Conclusion

Our current study provides new population-based evidence that in the early stages of these two diseases, hepatic steatosis may have a greater impact on T2D, compared to the opposite direction. Our findings also reveal that individuals having NAFLD face a significantly increased risk of T2D. Our exploration of the specific causal relationships between obesity, hepatic steatosis, and T2D would help to further elucidate the pathogenesis of NAFLD and identify the population at high risk for T2D. As supplement aggressive obesity control, targeting hepatic steatosis may be an alternative strategy for preventing T2D. Meanwhile, we recommend to pay more attention to the glycemic profile of patients with NAFLD.

### Electronic supplementary material

Below is the link to the electronic supplementary material.

Supplementary Material 1

## Data Availability

All data and materials that we used in the present study are available on reasonable request to the authors.

## Abbreviations

BFP: Body fat percentage
CFI: The comparative fitness index
CI: Confidence intervals
CMEC: China Multi-Ethnic Cohort
FBG: Fasting blood glucose
FLI: The fatty liver index
HR: Hazard Ratios
NAFLD: Nonalcoholic fatty liver disease
PDFF: Proton density fat fraction
SRMR: Standardized root mean square residual
T2D: Type 2 diabetes
