# Supplementary material for: Temporal relationship between hepatic steatosis and fasting blood glucose elevation: a longitudinal analysis from China and UK
Source: BMC Public Health. 2024 Jul 12;24:1865. doi: 10.1186/s12889-024-19177-3 (PMC11241918; doi:10.1186/s12889-024-19177-3)
Supplement: Supplementary file 1 — Supplementary Material 1 [file 12889_2024_19177_MOESM1_ESM.docx]

**Supplementary information**

**Temporal relationship between** **hepatic steatosis and fasting**

**blood glucose elevation: a longitudinal analysis from China and UK**

**Content**

[Figure. S1: The flow chart of the participants’ selection in CMEC and UK Biobank 1](#_Toc169365696)

[Figure. S2: The flow chart of the participants’ selection in survival analysis by FLI 2](#_Toc169365697)

[Figure. S3: The flow chart of the participants’ selection in validation analyses by PDFF 3](#_Toc169365698)

[Figure. S4: Schoenfeld residuals plots for the exposure (BFP)-outcome (type 2 diabetes) Cox proportional hazard model. 4](#_Toc169365699)

[Figure. S5: Schoenfeld residuals plots for the exposure (BFP)-mediator (FLI)-outcome (type 2 diabetes) Cox proportional hazard model 5](#_Toc169365700)

[Figure. S6: Schoenfeld residuals plots for the exposure (BFP) -mediator (PDFF)-outcome (type 2 diabetes) Cox proportional hazard model 6](#_Toc169365701)

[Table. S1: Baseline characteristics of participants with and without available data for the first repeat study. 7](#_Toc169365702)

[Table. S2: Descriptions of covariates incorporated in the two cohorts 9](#_Toc169365703)

[Table. S3: Test for exposure–mediator (FLI) interaction 14](#_Toc169365704)

[Table. S4: Test for exposure–mediator (PDFF) interaction. 15](#_Toc169365705)

[Table. S5: Cross-lagged path analysis of FLI with FBG in people who did not have NAFLD and type 2 diabetes at baseline 16](#_Toc169365706)

**
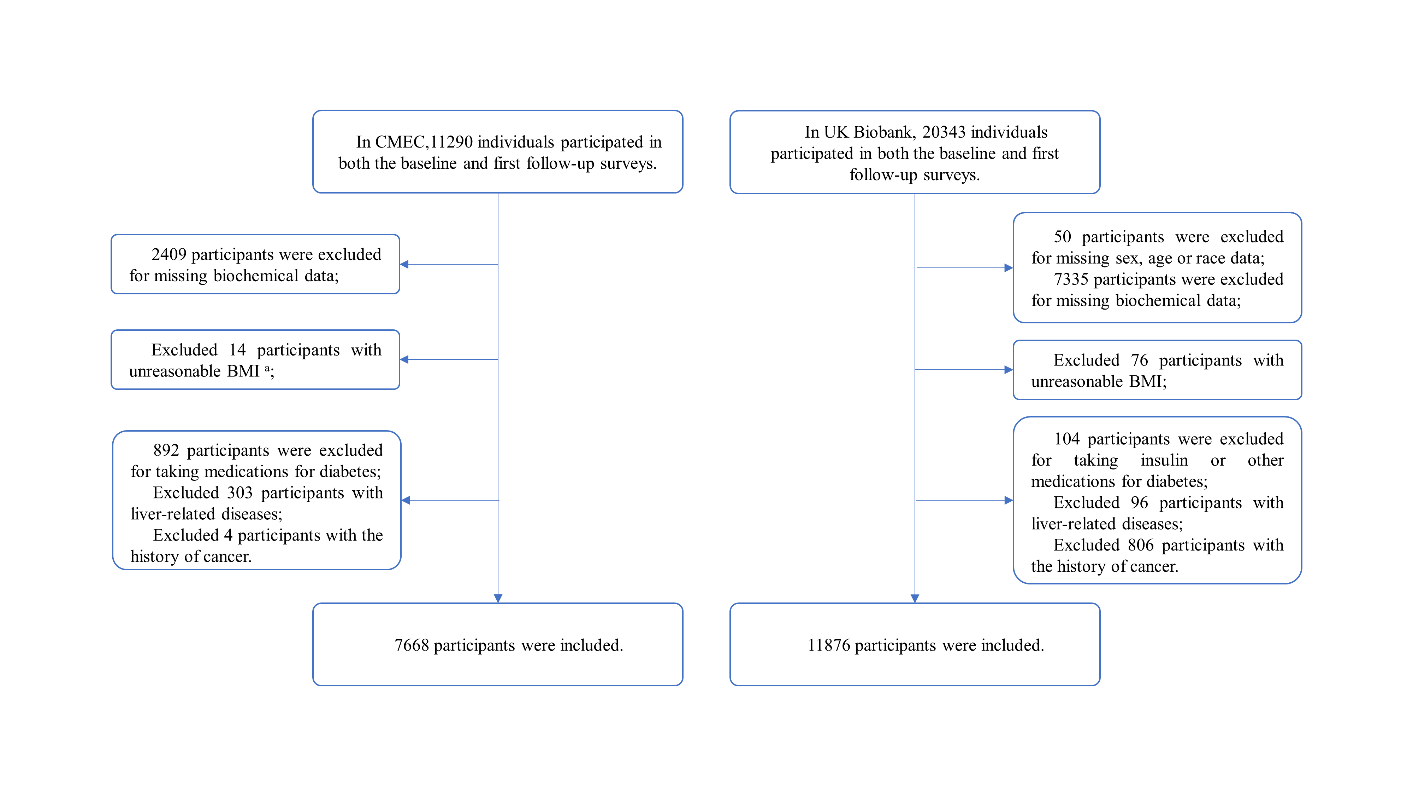
Figure. S1: The flow chart of the participants’ selection in CMEC and UK Biobank.**

^a^. Unreasonable BMI was defined as < 14 or > 45 kg/m^2^.

**Figure. S2: The flow chart of the participants’ selection in survival analysis by FLI.**

**
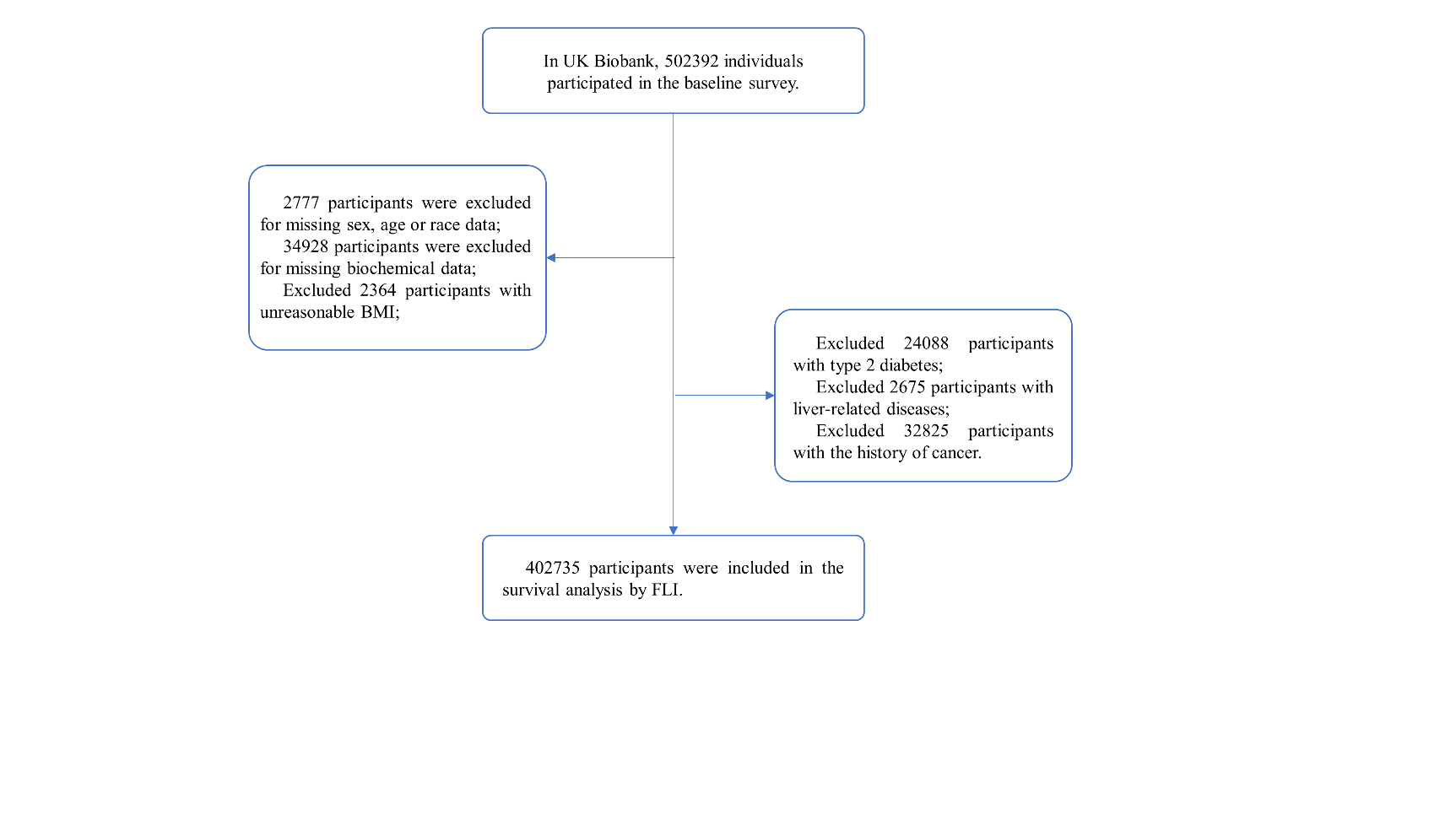
**

**
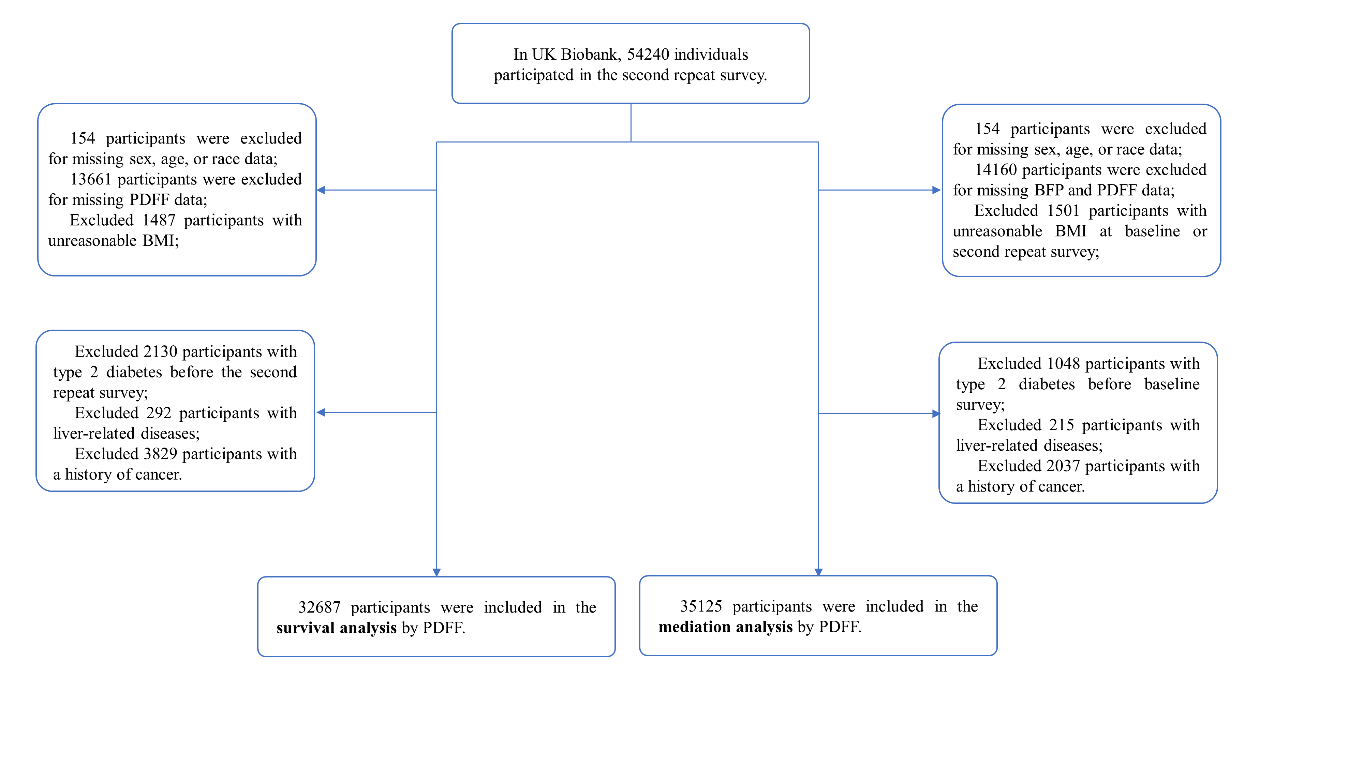
Figure. S3: The flow chart of the participants’ selection in validation analyses by PDFF.**

**
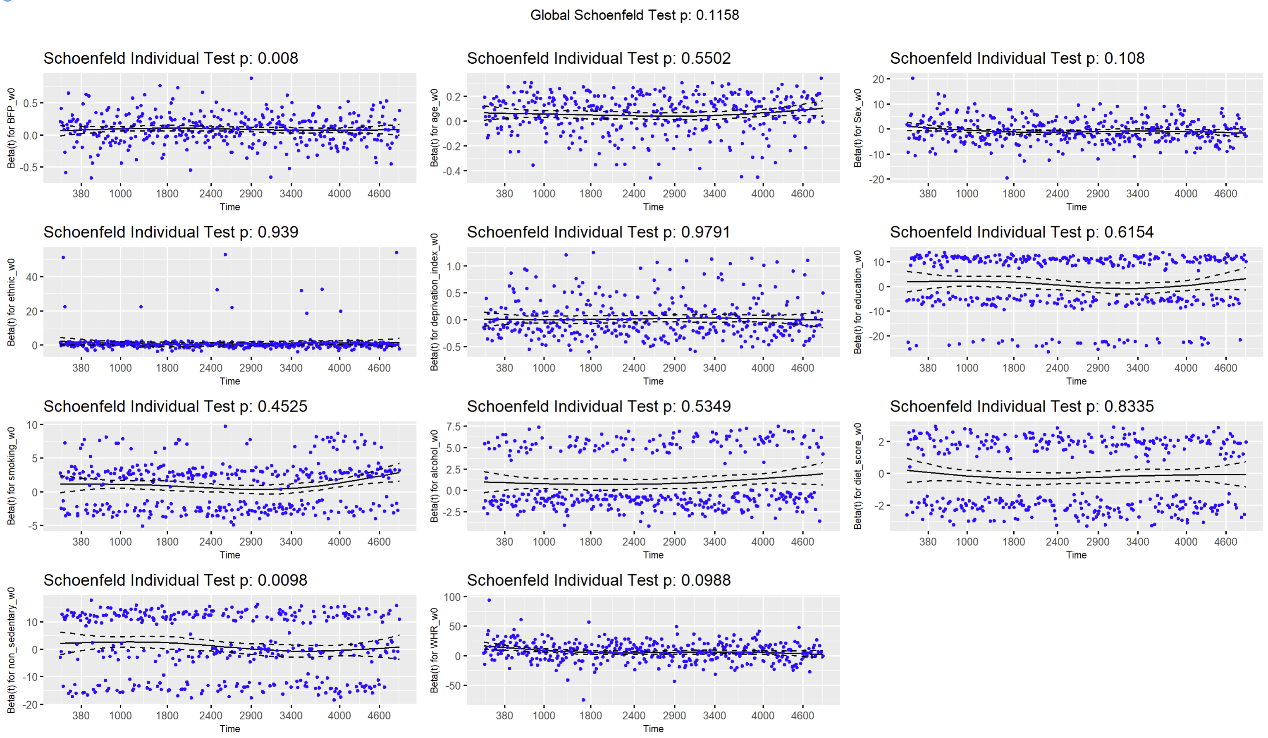
Figure. S4: Schoenfeld residuals plots for the exposure (BFP)-outcome (type 2 diabetes) Cox proportional hazard model ^a^.**

^a^. The global Schoenfeld test with a *P*-value greater than 0·05 indicates that the model satisfies the proportional hazard assumption. Although some individual variables had *P*-values less than 0·05, we do not consider them to have significantly violated the proportional hazard assumption due to the large sample size and the absence of time-dependent change patterns in the fitted lines.

**
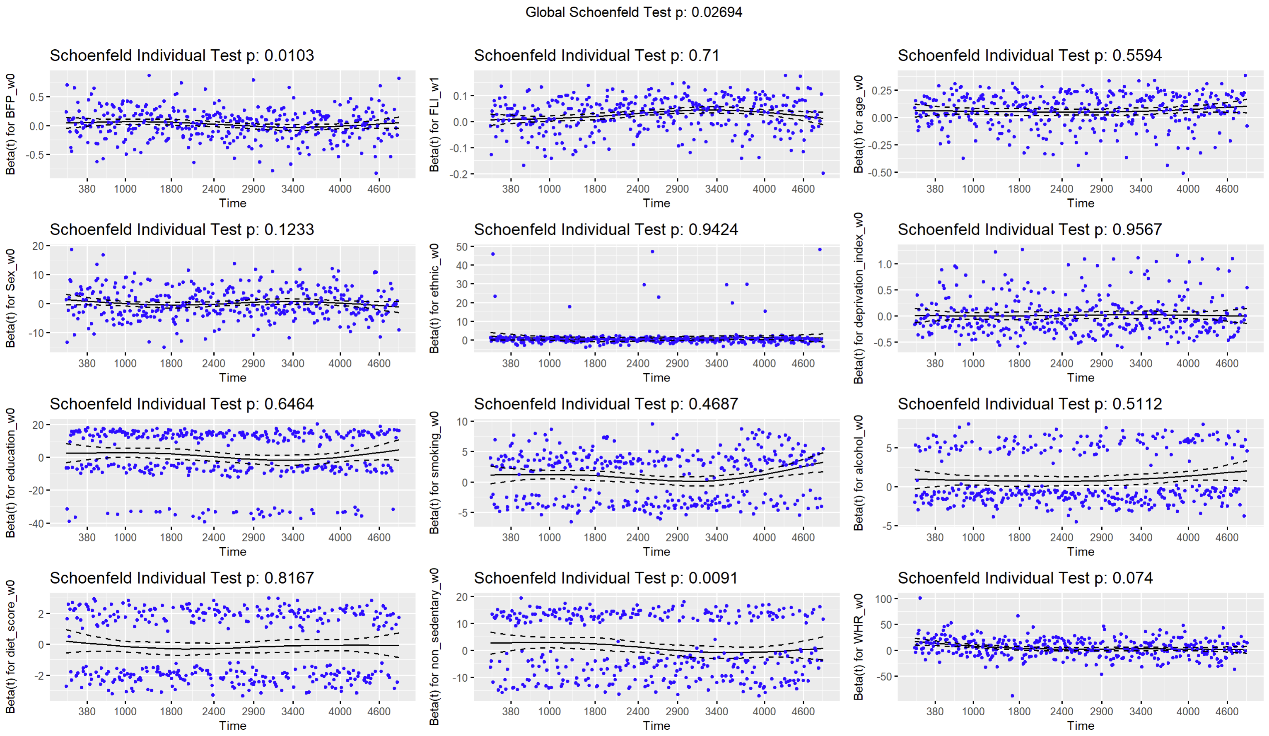
Figure. S5: Schoenfeld residuals plots for the exposure (BFP)-mediator (FLI)-outcome (type 2 diabetes) Cox proportional hazard model ^a^.**

^a^. The global Schoenfeld test with a *P*-value greater than 0·05 indicates that the model satisfies the proportional hazard assumption. Although some individual variables had *P*-values less than 0·05, we did not consider them to have significantly violated the proportional hazard assumption due to the large sample size and the absence of time-dependent change patterns in the fitted lines.

**
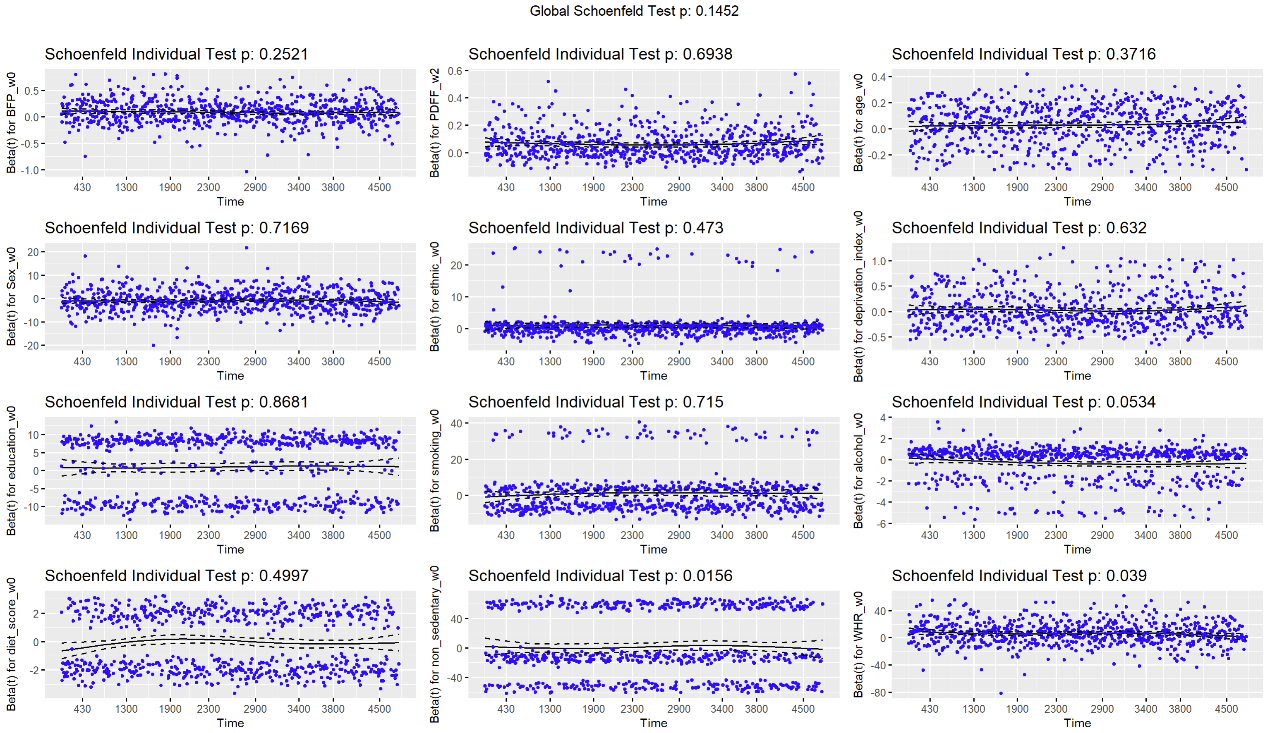
Figure. S6: Schoenfeld residuals plots for the exposure (BFP) -mediator (PDFF)-outcome (type 2 diabetes) Cox proportional hazard model ^a^.**

^a^. The global Schoenfeld test with a *P*-value greater than 0·05 indicates that the model satisfies the proportional hazard assumption. Although some individual variables had *P*-values less than 0·05, we do not consider them to have significantly violated the proportional hazard assumption due to the large sample size and the absence of time-dependent change patterns in the fitted lines.

**Table. S1: Baseline characteristics of participants with and without available data for the first repeat study** ^a^.

| **Characteristic** | **CMEC** | | |
| --- | --- | --- | --- |
|  | **Overall** | **Available for the first repeat study** | **Not available for the first repeat study** |
| **No. of participants** | 98631 | 11301 | 87330 |
| **Age (years)** | 51.00 (43.00, 60.00) | 51.00 (44.00, 59.00) | 50.00 (43.00, 60.00) |
| **Female** | 59762 (60.0) | 6857 (60.7) | 52905 (59.9) |
| **BMI (kg/m^2^)** | 24.04 (21.77, 26.31) | 24.34 (22.06, 26.64) | 24.01 (21.73, 26.30) |
| **Ethnicity, Majority (Han)** | 55443 (55.7) | 6844 (60.6) | 48599 (55.1) |
| **Education** |  |  |  |
| Less than high school | 77403 (77.7) | 8446 (74.7) | 68957 (78.1) |
| High school or equivalent | 11304 (11.4) | 1457 (12.9) | 9847 (11.2) |
| College or above | 10845 (10.9) | 1396 (12.4) | 9449 (10.7) |
| **Occupation** |  |  |  |
| Employed | 85593 (86.0) | 9643 (85.3) | 75950 (86.1) |
| Unemployed | 4332 (4.4) | 429 (3.8) | 3903 (4.4) |
| Retired | 9551 (9.6) | 1213 (10.7) | 8338 (9.4) |
| **Post-menopause in female** | 28578 (47.8) | 3219 (46.9) | 25359 (47.9) |
| **Current smoking** | 20142 (20.2) | 2039 (18.0) | 18103 (20.5) |
| **Drinking frequency** | | | |
| Current non-drinking | 57708 (58.0) | 6524 (57.7) | 51184 (58.0) |
| Occasional | 29118 (29.3) | 3424 (30.3) | 25694 (29.1) |
| Regular | 12695 (12.8) | 1352 (12.0) | 11343 (12.9) |
| **Current beverage consumption** ^b^ | 8058 (8.1) | 770 (6.8) | 7288 (8.3) |
| **Healthy diet** ^c^ | 32182 (32.3) | 4064 (36.0) | 28118 (31.9) |
| **Total energy intake (kcal/day)** | 1737.19 (1354.75, 2201.42) | 1751.27 (1378.22, 2219.88) | 1735.24 (1351.77, 2199.10) |
| **Physical activity (MET-hours/week)** | 153.60 (81.90, 260.15) | 153.00 (85.07, 254.80) | 153.71 (81.90, 260.69) |
| **Insomnia symptom** | 43695 (43.9) | 4969 (44.0) | 38726 (43.9) |
| **Fast blood glucose, mmol/L** | 5.12 [4.70, 5.56] | 5.14 [4.77, 5.56] | 5.11 [4.74, 5.56] |
| **Fatty liver index** | 23.89 [9.83, 49.86] | 24.91 [10.20, 51.67] | 23.77 [9.79, 49.63] |

^a^. Median [interquartile range] or counts (proportion).

^b^. In CMEC, beverage included sweeten beverages, coffee and caffeine beverages, and others.

^c^. A healthy diet is defined as a healthy diet score at the 60th percentile and above. In CMEC, a Dietary Approaches to Stop Hypertension (DASH) score was used.

**Table. S2: Descriptions of covariates incorporated in the two cohorts.**

| Covariates | Availability in two cohorts | Original questions | Types of covariates | Rules in the study | Descriptions | References |
| --- | --- | --- | --- | --- | --- | --- |
| Age | Both | “Date of birth” and “Date of the survey” in CMEC; “Date of birth” and “Date of attending assessment center” in UKB. | Continuous | Date of survey minus date of birth in years with two decimal places. | Age of participants at baseline and first repeated survey. |  |
| Sex | Both | “Sex” in both cohorts. | Dichotomous | Male, Female | Sex of participants. |  |
| Ethnicity/ Race | Both | “Ethnicity” in CMEC; “Ethnic background” in UKB. | Dichotomous | CMEC：Majority (Han), Minority (Dong, Bouyei, Yi, Miao, Bai, Tibetan)  UKB: Majority (White), Minority (Mixed, Other) | Ethnicity/ race of participants. The majority refers to Han Chinese in CMEC and refers to White in UKB. |  |
| Highest education | Both | “What is your highest level of education” in CMEC; “Which of the following qualifications do you have?” in UKB. | Multinomial | CMEC: No formal education, Less than high school, High school or equivalent, and College or above.  UKB: college or above (college or university degree); high school or equivalent (A levels, AS levels, or equivalent; O levels, GCSEs, or equivalent; CSEs or equivalent; NVQ, HND, HNC, or equivalent; other professional qualifications); less than high school (none of the above). | Highest level of education of the participants. |  |
| Occupation | CMEC only | “What is the type of your occupation” in CMEC. | Dichotomous | Employed, Unemployed | Current employment status of participants. |  |
| Townsend deprivation index, TDI | UKB only | - | Continuous | - | Townsend deprivation index calculated immediately prior to participant joining UKB. Based on the preceding national census output areas. Each participant is assigned a score corresponding to the output area in which their postcode is located. |  |
| Smoking | Both | “Do you smoke tobacco now?” in both cohorts. | Categorical | Current, Previous and Never | Participants' current smoking status, focusing on the behavior. |  |
| Non-sedentary physical activity | Both | Type and duration of occupational, traffic, chores, and leisure time activities in CMEC; “In a typical WEEK, on how many days did you do 10 minutes or more of moderate physical activities like carrying light loads, cycling at normal pace?” and “How many minutes did you usually spend doing moderate activities on a typical DAY?” in UKB. | Continuous | Physical activity considers participants’ occupational, traffic, chores, and leisure time activities. We then calculated each participant's hours of metabolic equivalent tasks per day (MET-h). | Participants in the CMEC were predominantly heavy laborers in the primary sector, and therefore had larger values for non-sedentary physical activity compared to the UKB. The values in CMEC are similar to those in other studies in China (e.g., CKB). |  |
| Dietary score | Both | Food Frequency Questionnaires (FFQ) in both cohorts. | Dichotomous | Healthy or Unhealthy | A healthy diet is defined as a healthy diet score at the 60th percentile and above in both cohorts. In CMEC, a Dietary Approaches to Stop Hypertension (DASH) score was used. In UKB, healthy diet score was calculated based on consumption of 7 dietary components. | Zhang Y, Yang H, Li S, Li WD, Wang Y. Consumption of coffee and tea and risk of developing stroke, dementia, and poststroke dementia: A cohort study in the UK Biobank. PLoS Med. 2021;18(11):e1003830.  Xiao X, Qin Z, Lv X, et al. Dietary patterns and cardiometabolic risks in diverse less-developed ethnic minority regions: results from the China Multi-Ethnic Cohort (CMEC) Study. The Lancet regional health Western Pacific 2021; 15: 100252. |
| Alcohol frequency | Both | “In the past year, about how often have you had a drink?” in CMEC; “About how often do you drink alcohol?” in UKB. | Categorical | Never, Occasional (less than three times a month), Regular (more than once a week) | Whether the participant has a current alcohol drinking. Answering any frequency of drinking is considered current drinking. |  |
| Waist-to-hip ratio WHR | Both | - | Continuous | WHR is obtained from the waist circumference divided by the hip circumference | WHR value here was constructed from waist circumference and hip circumference measured. |  |
| Self-reported hypertension | Both | “Have you been diagnosed with hypertension by a doctor in a hospital at township/district level or above?” in CMEC; “Has a doctor ever told you that you have had any of the following conditions?” in UKB. | Dichotomous | Yes or No | Participants' self-reported hypertension on the questionnaire. |  |
| Self-reported diabetes | Both | “Have you been diagnosed with diabetes by a doctor in a hospital at township/district level or above?” in CMEC; “Has a doctor ever told you that you have diabetes?” in UKB. | Dichotomous | Yes or No | Participants' self-reported diabetes on the questionnaire. |  |

**Table. S3: Test for exposure–mediator (FLI) interaction ^a^.**

| **Variable** | ***β*** | ***SE*** | ***P*** |
| --- | --- | --- | --- |
| Exposure: BFP | -0·0134 | 0·0245 | 0·5792 |
| Mediator: FLI | 0·0120 | 0·0089 | 0·1784 |
| BFP-FLI interaction | 0·0005 | 0·0002 | 0·0785^b^ |

Abbreviation: BFP: body fat percentage; FLI: the fatty liver index.

^a^. The exposure–mediator interaction test model was constructed as: $\log h\left( t \right)=\log h_{0}\left( t \right)+\beta_{1}exposure+\beta_{2}mediator+\beta_{3}exposure*mediator+\beta_{4}^{'}C$, in which $h\left( t \right)$ is the hazard rate of developing type 2 diabetes. $C$ in models included age, sex, waist-to-hip ratio, ethnicity, deprivation index, education, smoking status, alcohol consumption, dietary score, and non-sedentary physical activity.

^b^. *P*-value for BFP-FLI interaction coefficient greater than 0·05, we considered that there is no interaction effect between BFP and FLI.

**Table. S4: Test for exposure–mediator (PDFF) interaction ^a^.**

| **Variable** | ***β*** | ***SE*** | ***P*** |
| --- | --- | --- | --- |
| Exposure: BFP | 0.0492 | 0.0065 | <0.0001 |
| Mediator: PDFF | 0.0732 | 0·0176 | <0.0001 |
| BFP-PDFF interaction | -0.0003 | 0·0005 | 0.5616 ^b^ |

Abbreviation: BFP: body fat percentage; PDFF: Proton density fat fraction.

^a^. The exposure–mediator interaction test model was constructed as: $\log h\left( t \right)=\log h_{0}\left( t \right)+\beta_{1}exposure+\beta_{2}mediator+\beta_{3}exposure*mediator+\beta_{4}^{'}C$, in which $h\left( t \right)$ is the hazard rate of developing type 2 diabetes. $C$ in models included age, sex, waist-to-hip ratio, ethnicity, deprivation index, education, smoking status, alcohol consumption, dietary score, and non-sedentary physical activity.

^b^. *P*-value for BFP-PDFF interaction coefficient greater than 0·05, we considered that there is no interaction effect between BFP and PDFF.

**Table. S5: Cross-lagged path analysis of FLI with FBG in people who did not have NAFLD and type 2 diabetes at baseline ^a^.**

|  | **CMEC** | |  | **UK Bio-bank** | |
| --- | --- | --- | --- | --- | --- |
|  | ***β* _CMEC_** | ***P*** |  | ***β*** **_UK Bio-bank_** | ***P*** |
| **Without two diseases** | | | | | |
| Baseline FBG 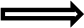 Follow-up FLI | 0·027 | 0·004 |  | 0·004 | 0·587 |
| Baseline FLI 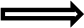 Follow-up FBG | 0·065 | < 0·001 |  | 0·044 | < 0·001 |
| *P*-value ^b^ | < 0·01 | - |  | < 0·01 | - |
| Abbreviation: FLI: the fatty liver index; FBG: fast blood glucose; NAFLD: non-alcoholic fatty liver disease; CMEC: China Multi-Ethnic cohort. | | | | | |
| ^a^. In these two models, we adjusted for age, sex, ethnicity, waist-hip ratio, occupation or deprivation index, education, smoking status, alcohol consumption, dietary score, and non-sedentary physical activity. These two models fitted well with comparative fitness index (CFI) ≥ 0·95 and standardized root mean square residual (SRMR) ≤ 0·08. | | | | | |
| ^b^. *P*-value reflected the difference between two path coefficients *β* using Fisher's Z test. | | | | | |
